# Novel Insights into Mice Multi-Organ Metabolism upon Exposure to a Potential Anticancer Pd(II)-Agent

**DOI:** 10.3390/metabo11020114

**Published:** 2021-02-17

**Authors:** Tatiana J. Carneiro, Rita Araújo, Martin Vojtek, Salomé Gonçalves-Monteiro, Carmen Diniz, Ana L. M. Batista de Carvalho, M. Paula M. Marques, Ana M. Gil

**Affiliations:** 1 Department of Chemistry and CICECO—Aveiro Institute of Materials, University of Aveiro, 3810-193 Aveiro, Portugal; tatiana.joao@ua.pt (T.J.C.); anarita.asilva@ua.pt (R.A.); 2 LAQV/REQUIMTE, Laboratory of Pharmacology, Department of Drug Sciences, Faculty of Pharmacy, University of Porto, 4050-313 Porto, Portugal; matovoj@gmail.com (M.V.); salomegmonteiro@hotmail.com (S.G.-M.); carmen.diniz@gmail.com (C.D.); 3 R&D Unit “Molecular-Physical Chemistry”, University of Coimbra, 3004-535 Coimbra, Portugal; analmbc@gmail.com (A.L.M.B.d.C.); pmc@ci.uc.pt (M.P.M.M.); 4 Department of Life Sciences, Faculty of Science and Technology, University of Coimbra, 3000-456 Coimbra, Portugal

**Keywords:** palladium(II)-drugs, Pd_2_Spm, cisplatin, mice, NMR metabolomics, tissue extracts

## Abstract

Pd(II)-compounds are presently regarded as promising anticancer drugs, as an alternative to Pt(II)-based drugs (e.g., cisplatin), which typically trigger severe side-effects and acquired resistance. Dinuclear Pd(II) complexes with biogenic polyamines such as spermine (Pd_2_Spm) have exhibited particularly beneficial cytotoxic properties, hence unveiling the importance of understanding their impact on organism metabolism. The present study reports the first nuclear magnetic resonance (NMR)-based metabolomics study to assess the *in vivo* impact of Pd_2_Spm on the metabolism of healthy mice, to identify metabolic markers with possible relation to biotoxicity/side-effects and their dynamics. The changes in the metabolic profiles of both aqueous and lipophilic extracts of mice kidney, liver, and breast tissues were evaluated, as a function of drug-exposure time, using cisplatin as a reference drug. A putative interpretation was advanced for the metabolic deviations specifically triggered by Pd_2_Spm, this compound generally inducing faster metabolic response and recovery to control levels for all organs tested, compared to cisplatin (except for kidney lipid metabolism). These results constitute encouraging preliminary metabolic data suggestive of potential lower negative effects of Pd_2_Spm administration.

## 1. Introduction

The anticancer properties of Pt(II)-containing compounds were first revealed upon the serendipitous discovery of cisplatin’s (*cis*-Pt(NH_3_)_2_Cl_2_, cDDP) antiproliferative effects [1,2]. Since the approval of this drug as a chemotherapeutic agent in 1978, this complex has been successfully used to treat several solid tumors [3,4]. However, its high toxicity and acquired resistance are recognized drawbacks to its application in oncology [5,6], which have prompted intense research on biologically active alternative cDDP-analogs, e.g., carboplatin and oxaliplatin, also approved for use in clinical practice [7,8,9]. Although the latter compounds seem to exhibit lower nephrotoxicity and ototoxicity [6,10], in many cases, they are associated with (i) hematologic and cardiac toxicity [11] and (ii) lower therapeutic effectiveness, compared to cisplatin [6,12]. Indeed, the toxic side effects of Pt(II) compounds are known to be accompanied by a significant impact on intracellular function (e.g., DNA damage, organelle dysfunction, oxidative stress, and inflammation) [3,13,14], effects which may be responsible for deviations in *in vivo* metabolic status, as observed by multi-organ metabolomics, as previously reported for cisplatin [15].

The deleterious effects of Pt-drugs have triggered the development of complexes [9,16] with transition-metal centers such as palladium(II), gold(I)/(III), and ruthenium(II)/(III) [8,17,18]. Pd(II) has been recognized as one of the best candidates due to its physical-chemical similarities to Pt(II) [8,14]. Indeed, several Pd(II) complexes have been tested *in vitro* for anti-tumor properties in breast [19,20,21,22,23], lung [24,25], colon [26,27,28], prostate [29,30,31], gastric [32], liver [33], and other human cancer cell lines [34,35,36,37]. In some cases, favorable antiproliferative and cytotoxic properties have been reported for Pd(II) agents [20,24,28], compared to Pt(II) analogs, along with antimetastatic properties (antiangiogenic and anti-migratory [22]), lower acquired resistance [36], and lesser toxicity towards non-neoplastic cells [20]. Favorable ligands to soft-ions such as Pt(II) and Pd(II) comprise biogenic polyamines (PA) [26,27], e.g., putrescine (H_2_N(CH_2_)_4_NH_2_, Put), spermidine (H_2_N(CH_2_)_4_NH(CH_2_)_3_NH_2_, Spd) and spermine (H_2_N(CH_2_)_3_NH(CH_2_)_4_NH(CH_2_)_3_NH_2_, Spm), all essential polycations impacting on eukaryotic cell growth and differentiation [38]. The resulting polynuclear chelates have been shown to target cellular DNA (at concentrations as low as 5 μg/mL) [39,40] through non-conventional interactions (inter-strand, long-range) [41,42], thus inducing significant cytotoxicity against several types of human cancer cells [19,20,21,35,36,43]. Indeed, PAs form stable, flexible, and soluble complexes with Pd(II) [8,36], improving diffusion across cell membranes [35,40] and, therefore, promoting higher bioavailability of the complex to cancer cells [40]. Recent studies have focused on the biological performance of Pt/Pd complexes with PA regarding: (i) metal center (either Pt(II) or Pd(II)) [21,35,36,44] and (ii) type of ligand (either Spd or Spm) [39,40]. In particular, the antitumor properties of dinuclear Pd(II) complexes with biogenic polyamines such as spermine (Pd_2_Spm) have been studied mainly *in vitro*, in human cell lines of oral squamous carcinoma (HSC-3) [35], ovarian carcinoma (A2780) [36], osteosarcoma [44], and breast cancer (MCF-7 [19] and MDA-MB-231 [19,21,22]), generally exhibiting a tendency for enhanced cytotoxicity, compared to cDDP and its Pt(II) analog [35,36].

New knowledge on the impact of Pd(II)-complexes on cell and organism metabolism is needed for a better understanding of the mode of action of these potential drugs. In recent years, several untargeted metabolic studies have been carried out mostly *in vitro* (e.g., addressing breast and prostate cancer cell lines) and sometimes compared to *in vivo* behavior (murine models), as recently reviewed [45]. Such studies evidenced metabolic effects associated with mechanisms of induced cell death and antioxidant defense, disclosing deviations in the levels of reactive oxygen species (ROS), and specific metabolites related to mitochondrial respiration and antioxidant defense (e.g., ATP and glutathione) [45]. Further metabolic perturbations produced *in vitro* and *in vivo* (both locally (tissues) and systemically (biofluids)) are detectable by metabolomics and may pave the way for defining possible new markers of drug toxicity, efficacy, or resistance [46,47]. Unlike for cisplatin [48,49,50], the effects of Pd(II)-complexes have rarely been evaluated by metabolomics [16], to the best of our knowledge, with a single report comprising the impact of Pd_2_Spm complex on human osteoblasts (HOb) and osteosarcoma (MG-63) cells using nuclear magnetic resonance (NMR)-based metabolomics [51].

To our knowledge, this is the first ^1^H NMR metabolomics study aimed at assessing the *in vivo* impact of Pd_2_Spm on the metabolism of healthy mice as a possible source of markers of toxicity and/or secondary effects. This was carried out by NMR metabolic profiling of kidney and liver tissues (two of the organs known to be most affected by cDDP toxicity [52]), as well as breast tissue (potentially contributing to future studies of breast cancer). This work compared magnitude and dynamics (in a 1–48 h period) of the impact of Pd_2_Spm on the metabolome of each organ (compared to cDDP), attempting to establish relative timescales of metabolic cellular response and recovery.

## 2. Results

### 2.1. ^1^H NMR Spectra of Aqueous and Lipophilic Metabolomes of Mouse Kidney, Liver, and Breast Tissue

The representative ^1^H NMR spectra of aqueous extracts of control murine kidney, liver, and breast tissue (not shown) have been reported before [15], having shown expected relatively high levels of betaine, *m*-inositol, and inosine in the kidney; of acetone, glucose, glycogen and reduced glutathione (GSH) in the liver; and of creatine, phosphocholine (PC) and glycerolipids in breast tissue. In relation to the ^1^H NMR spectra of the lipophilic extracts of the same tissue types (Figure S1), the main differences are in agreement with previous reports [53,54,55] and comprised: (1) decreasing cholesterol levels from kidney to liver tissue, with no detectable cholesterol moieties in breast tissue; (2) relatively higher phosphatidylcholine (PTC) levels in the liver; with hardly any detectable phospholipids in breast tissue; and (3) significantly higher levels of glycerolipids in breast tissue, compared to kidney and liver tissue.

### 2.2. Impact of Pd_2_Spm on Kidney Metabolome

**Figure 1:**
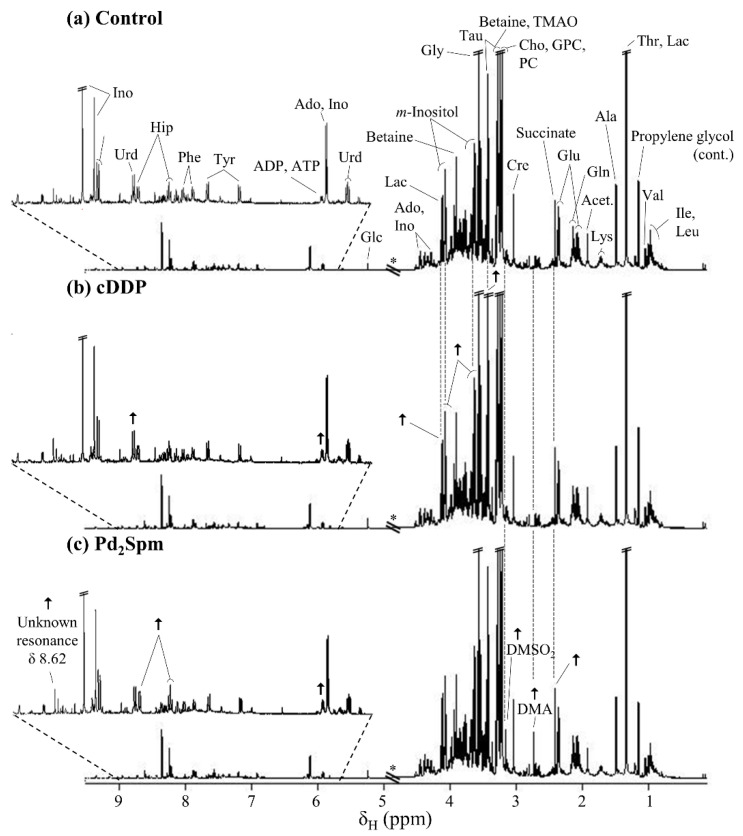
Average 500 MHz ^1^H NMR spectra of aqueous kidney extracts of BALB/c mice after 1 h of post-injection with (**a**) phosphate buffer saline solution (controls), (**b**) cisplatin (cDDP), and (**c**) dinuclear Pd(II) complex with spermine (Pd_2_Spm). * Cut-off spectral region due to water suppression (δ 4.53–5.20); cont.: contamination. Arrows represent noticeable metabolite variations in drug-exposed samples compared to controls. Dashed lines are used to guide the eye. Metabolite abbreviations: three-letter code used for amino acids; Acet., acetate; Ado, adenosine; ADP, adenosine diphosphate; ATP, adenosine triphosphate; Cho, choline; Cre, creatine; DMA, dimethylamine; DMSO_2_, dimethyl sulfone (tentative assignment); Glc, glucose; GPC, glycerophosphocholine; Hip, hippurate; Ino, inosine; Lac, lactate; PC, phosphocholine; Tau, taurine; TMAO, trimethylamine-*N*-oxide; Urd, uridine.

Visual inspection of the average spectra of kidney polar extracts, after 1 h exposure to each drug (Figure 1), suggested apparent Pd_2_Spm-specific spectral changes, such as increases in succinate, dimethylamine (DMA), dimethylsulfone (DMSO_2_) (tentative assignment), ADP/ATP, hippurate, and a singlet resonance at δ 8.62.

**Figure 2:**
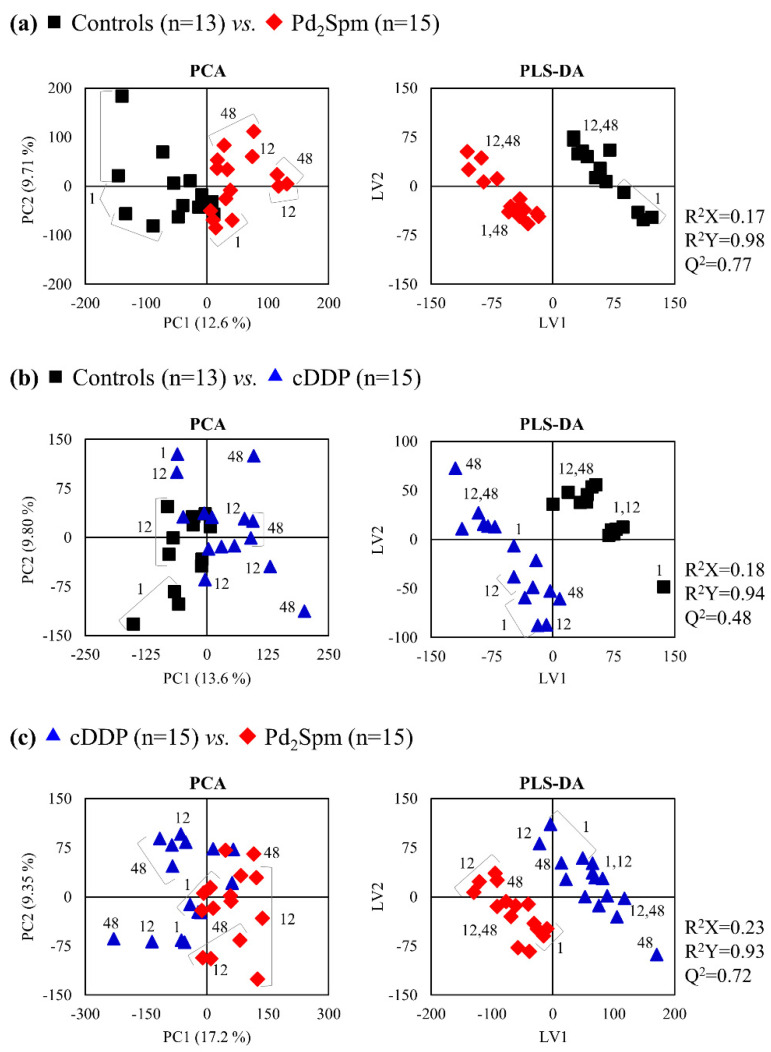
Principal component analysis (PCA) (**left**) and partial least squares-discriminant analysis (PLS-DA) (**right**) scores scatter plots for ^1^H nuclear magnetic resonance (NMR) spectra of aqueous extracts of all time-course samples of kidney of BALB/c mice: (**a**) Pd_2_Spm-exposed (*n* = 15, red diamonds) and controls (*n* = 13, black squares), (**b**) cDDP-exposed (*n* = 15, blue triangles) and controls (*n* = 13, black squares), and (**c**) Pd_2_Spm- and cDDP-exposed (*n* = 15 in each group, symbols as indicated above). Numbers represent time-points of drug exposure in hours. Validation metrics (R^2^ and Q^2^ values) are specified for each model.

However, a more objective assessment of significant spectral variations requires evaluation by multivariate and univariate statistics. Considering all exposure times, the effect of the Pd_2_Spm complex (Figure 2a) seemed to be stronger than that of cDDP (Figure 2b), defining a clearer group separation in principal component analysis (PCA) and providing a more robust partial least squares-discriminant analysis (PLS-DA) model, with predictive power (Q^2^) of 0.77, compared to cDDP (Q^2^ 0.48). Furthermore, the important separation of the two drug-treated mouse groups (Figure 2c, Q^2^ 0.72) indicated that distinct kidney metabolic responses occur for the different complexes.

**Figure 3:**
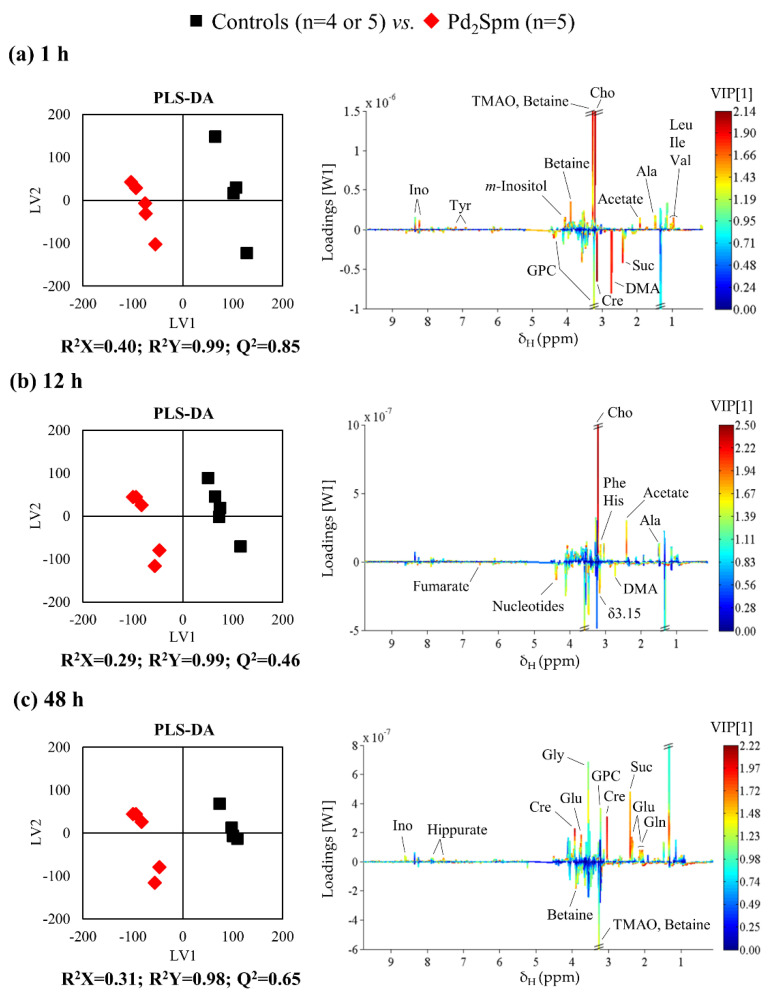
Pairwise PLS-DA scores scatter plots (**left**) and LV1 loadings plots (**right**) obtained for ^1^H NMR spectra of aqueous kidney extracts of BALB/c mice at (**a**) 1 h, (**b**) 12 h and (**c**) 48 h post-injection with Pd_2_Spm (red diamonds), compared to controls (black squares). Validation metrics (R^2^ and Q^2^ values) are specified for each pairwise comparison. Main peak assignments are indicated in loadings plots (Suc: succinate, and other compound abbreviations as in Figure 1 caption) and colored according to variable importance to projection (VIP).

Pairwise PCA and PLS-DA analysis performed for each drug and exposure time provided predictive power values, which, when closer to one, are indicative of higher strength and number of metabolite changes induced by each drug on the organ metabolic profile. For kidney tissue, such results (Table 1) indicated that polar metabolome responds strongly to both complexes upon 1 h of exposure, with a subsequent gradual decrease in predictive power for cDDP (reflecting gradually fewer differences compared to controls, thus, probably indicative of some degree of recuperation). A distinct time dependence was suggested for Pd_2_Spm, with some attenuation of Q^2^ at 12 h (possibly due to partial recuperation), and an apparent subsequent stronger group separation at 48 h (Table 1 and Figure 3). Based on the highlighted peaks in the corresponding latent variable 1 (LV1) loadings (Figure 3, right), selected peaks were integrated and subjected to univariate analysis, the results (Table S1) revealing several metabolite variations in common with those induced by cDDP, whereas a significant number of statistically relevant Pd_2_Spm-specific variations were identified (^b^ in Table S1).

**Figure 4:**
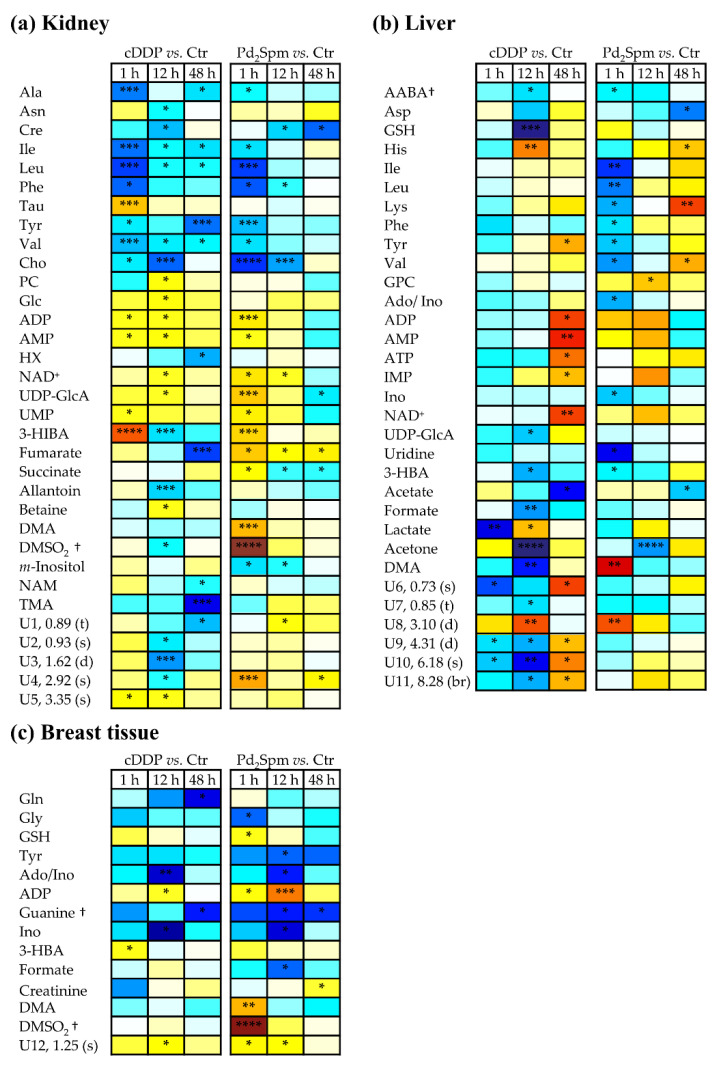
Heatmap illustrating the metabolic variations of polar compounds of (**a**) kidney, (**b**) liver, and (**c**) breast tissue of BALB/c mice at 1 h, 12 h, and 48 h post-injection with cDDP or Pd_2_Spm, compared to controls. The scale is color-coded as a function of Effect Size, from minimum (dark blue) to maximum (dark red) values. † Tentative assignment. Abbreviations: Ctr., controls; 3-letter code used for amino acids; 3-HBA, 3-hydroxybutyrate; 3-HIBA, 3-hydroxyisobutyrate; AABA, 2-aminobutyrate; Ado, adenosine; ADP, adenosine diphosphate; AMP, adenosine monophosphate; ATP, adenosine triphosphate; Cho, choline; Cre, creatine; DMA, dimethylamine; DMSO_2_, dimethyl sulfone; Glc, glucose; GSH, reduced glutathione; HX, hypoxanthine; IMP, inosine monophosphate; Ino, inosine; PC, phosphocholine; Tau, taurine; TMA, trimethylamine; NAD^+^, nicotinamide adenine dinucleotide; NAM, niacinamide; UDP-GlcA, urdine-diphosphate- glucuronate; Ui, unassigned i; UMP, uridine monophosphate. *: *p*-value < 5.0 × 10^−2^; **: *p*-value < 1.0 × 10^−2^; ***: *p*-value < 1.0 × 10^−3^; ****: *p*-value < 1.0 × 10^−4^, s: singlet, d: doublet, t: triplet, br: broad signal.

The heatmap form of Table S1 (Figure 4a) illustrated that, overall, the main features noted are: (1) amino acids-while both agents induced significant change (mostly decreases) in amino acids early on, Pd_2_Spm did not affect taurine levels, contrary to cDDP (1 h); (2) choline compounds-Pd_2_Spm left PC levels mostly unchanged, also contrary to cDDP; (3) nucleotides and derivatives-Pd_2_Spm induced stronger increases in ADP, NAD^+^ and UDP-GlcA, followed by a qualitative depletion at 48 h, suggestive of faster recuperation of nucleotide levels, with the exception of UDP-GlcA which remained significantly lower than controls (effect again not noted with cDDP); (4) Krebs cycle intermediates-fumarate and succinate followed a clearly distinct Pd_2_Spm-induced pattern, compared to cDDP, (5) additional distinct features of Pd_2_Spm impact related to DMA (inc.), DMSO_2_ (inc.), *m*-inositol (dec.) and lack of significant changes in allantoin, betaine, NAM and TMA (which were clearly observed with cDDP).

**Figure 5:**
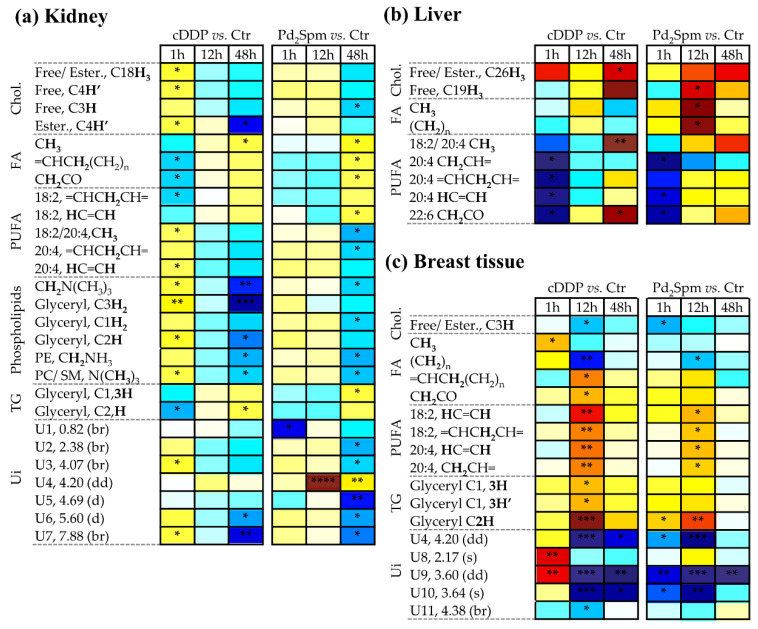
Heatmap illustrating the metabolic variations of lipophilic compounds of (**a**) kidney, (**b**) liver, and (**c**) breast tissue of BALB/c mice at 1 h, 12 h, and 48 h post-injection with cDDP or Pd_2_Spm, compared to controls. The scale is color-coded as a function of Effect Size, from minimum (dark blue) to maximum (dark red) values. Protons represented in bold correspond to the specific indicated assignment. Abbreviations: Chol., cholesterol; Ester., esterified; FA, fatty acids (general); PTC, phosphatidylcholine; PE, phosphatidylethanolamine; PUFA, poly-unsaturated fatty acid; SM, sphingomyelin; TG, triacylglycerides; Ui, unassigned i. *: *p*-value < 5.0 × 10^−2^; **: *p*-value < 1.0 × 10^−2^; ***: *p*-value < 1.0 × 10^−3^; ****: *p*-value < 1.0 × 10^−4^, dd: doublet of doublets. Other abbreviations as in Figure 4 caption.

In relation to the kidney lipophilic metabolome (Figure S2), the impact of exposure, as viewed by Q^2^ values (Table 1), appeared to be maximum at 48 h for both complexes, following a slightly distinct time-course dependence for each drug. Considering variations in specific metabolites (Table S4 and Figure 5a), Pd_2_Spm hardly affected the metabolic profile of kidney at 1 h (contrary to cDDP, which delivered an early strong impact on kidney lipophilic metabolites), then inducing a set of significant changes at 48 h, in broad agreement with those observed with cDDP, namely tendencies for decreased cholesterol species, lower phospholipids, and higher triglycerides content. In relation to fatty acids (FA), variations were noted mainly in unsaturated FA (significant with the Pd complex, and not with cDDP) (Figure 5a), with more enhanced Pd_2_Spm-induced decreases in average chain length ([CH_2_]_n_/CH_3_) and unsaturation degree (CH=CH/CH_3_) (Table S4).

### 2.3. Impact of Pd_2_Spm on Liver Metabolome

In relation to the liver metabolome, multivariate analysis (Table 1) indicated that the liver polar metabolome (Figure S3) responded more strongly and rapidly (at 1 h, Q^2^ 0.68) to Pd_2_Spm, compared to cDDP (Q^2^ 0.42), possibly showing signs of earlier recuperation at 48 h (with Q^2^ 0.33, compared to 0.52 for cDDP). Indeed, Pd_2_Spm induced an early strong decrease in amino acids (Table S2 and Figure 4b), change, which was partially reversed from 12 h onwards, with persisting decreased aspartate and increased histidine, lysine, and valine at 48 h (such changes are only qualitatively suggested for cDDP). In addition, Pd_2_Spm also induced a significant increase of DMA at 1 h, contrary to cDDP. Furthermore, Pd_2_Spm does not induce significant changes in liver nucleotides, whereas cDDP increased the levels of ADP, AMP, ATP, and inosine monophosphate (IMP) significantly at 48 h. Finally, changes in other compounds generally follow similar effects (although these tend to become stronger and more significant with cDDP).

In relation to the liver lipophilic metabolome, the Pd_2_Spm complex seemed to induce weaker or no effects in the 1–48 h period (Table 1), whereas cDDP resulted in strong effects at extreme times (1 h and 48 h) (Table 1 and Table S4). In particular, both drugs induced small cholesterol increases and an early (1 h) use of polyunsaturated fatty acids (PUFA) (more pronounced for cDDP), which were reversed in both cases in the 12–48 h period (Figure 5b). Notably, 18:2/20:4 and 22:6 moieties remain more elevated than controls at 48 h, in both cases (although only significant for cDDP). However, an apparent Pd_2_Spm-specific effect related to an increase in saturated FA at 12 h.

### 2.4. Impact of Pd_2_Spm on Breast Tissue Metabolome

For breast tissue, multivariate analysis (Table 1) indicated a more immediate impact of Pd_2_Spm on the polar metabolome (Q^2^ 0.43 at 1 h), compared to cDDP (Q^2^ < 0). In addition, the Pd complex did not seem to affect lipophilic extracts (Q^2^ < 0.5) as significantly as cDDP (Q^2^ > 0.5 for 1 and 12 h). However, both compounds induced some changes up to 12 h, and fewer changes at 48 h (evidencing a tendency for recuperation, which seemed more effective with Pd_2_Spm (Q^2^ ≈ 0).

The changes in polar metabolites (Table S3 and Figure 4c) were qualitatively similar for both drugs (although often varying in statistical significance), namely regarding the following metabolites: 1) glutamine, glycine, GSH, and tyrosine, 2) Ado/Ino, ADP (more enhanced upon Pd_2_Spm exposure), guanine (tentative assignment), 3-HBA, creatinine and two unassigned resonances (Figure 4c). Differences specific to Pd_2_Spm, compared to cDDP, comprised depletion in formate levels at 12 h, and marked increases in DMA and DMSO_2_ at 1 h.

In relation to the lipophilic metabolome of breast tissue (Table S4 and Figure 5c), both drugs induced a relative increase in unsaturated FA, compared to saturated ones (the latter viewed by the (CH_2_)_n_ resonance), although Pd_2_Spm-induced changes were less marked in relation to cDDP, consistently with the relatively lower Q^2^ values of PLS-DA models (Table 1). Unassigned resonances U8 (singlet at δ 2.17) and U9 (doublet of doublets at δ 3.60) were also distinguishable for both drugs, in terms of their variation, particularly at 1 h. In addition, both drugs induced a decrease in average chain length and unsaturation degree of FAs in the 1–12 h period, recovering to control levels at 48 h (Table S4).

## 3. Discussion

Of the three organs under study, the kidney was the most strongly affected by Pd_2_Spm, at both polar and lipophilic metabolome levels, exhibiting a distinct dynamic response, compared to cDDP. Indeed, the Pd complex enabled a clearer recovery of the kidney polar metabolome at 48 h, although deviant behavior remained affecting the tricarboxylic acid (TCA) cycle (fumarate excess and succinate depletion), suggesting enhanced activation of glycosylation (UDP-GlcA depletion) and creatine (depletion) pathways. Similarly to cDDP, Pd_2_Spm decreased amino acid levels in kidney tissue (reabsorption impairment of amino acids in kidney tissue due to nephrotoxicity [15,56] and, thus, inducing increased amino acid excretion [49]), and impacts on nucleotide metabolism (although more strongly); however, contrary to cDDP, such deviations were almost completely recovered to control levels at 48 h. Furthermore, Pd_2_Spm affected membrane metabolism differently (no PC changes, along with strong choline depletion featuring in common to both drugs), while not affecting the levels of allantoin, betaine, taurine, NAM, and TMA, all of which have been reported to change significantly upon cDDP exposure [15]. Instead, Pd_2_Spm specifically increased DMA and DMSO_2_, and decreased *m*-inositol, early on (1 h) (contrary to cDDP). The higher consumption of the latter may relate to its possible role in protective antioxidative mechanisms, although no changes could be observed in the GSH/GSSG system (only GSH was detected and at residual levels). As to the strong DMA and DMSO_2_ increases, we suggest that they may be considered as early markers of kidney response to the Pd_2_Spm complex, possibly reflecting the strong choline usage towards betaine synthesis in gut microbiota metabolism, which may: (1) lead to DMA synthesis through TMA or TMAO [57] (the latter detected in this study, following a weak decreasing tendency); and (2) originate methionine and enter sulfur metabolism, related to gut microbiota, originating dimethylsulfide (DMS) and dimethylsulfoxide (DMSO), in turn, oxidized to DMSO_2_ [58]. Both of these pathways seemed to be particularly active in kidney tissue upon Pd_2_Spm administration, as compared to cDDP. The possibility that dissolution of Pd_2_Spm in 1% DMSO (see Materials and Methods) may contribute to increased DMSO_2_ levels can not be ruled out at this stage, although a low (but not determined) DMSO concentration is expected to reach mouse tissues. Furthermore, a control experiment using DMSO alone in the same concentration as used for Pd_2_Spm dissolution, which would have been interesting, was hindered by ethical constraints. In any case, the relevance of this particular pathway was confirmed as it was also affected (although differently) in cDDP-treated animals where DMSO was not employed (DMSO_2_ decrease at 12 h, Table S1). The response of kidney lipid metabolism to Pd_2_Spm was delayed, compared to cDDP, and it presented an exception to the general behavior of fast response and recovery for Pd_2_Spm-exposed mice. In addition, the Pd(II) complex specifically lead to the biosynthesis of shorter and less unsaturated FA as part of a lipidic profile, which, as with cDDP, showed no signs of recuperation at 48 h. These metabolic characteristics in kidney tissue, in response to Pd_2_Spm, may potentially be related to a different degree of associated nephrotoxicity, although this hypothesis requires further investigation, as no biochemical measurements of biotoxicity were available in this work, as also noted in the related report dedicated to cDDP [15].

The dynamics of liver response to Pd_2_Spm were similar to that of kidney tissue (particularly in relation to polar metabolites), in that it seems to be shorter-term (1 h, vs. 12–48 h), and they recovered more rapidly, compared to cDDP. Faster (1 h) consumption of hepatic amino acids was triggered by the Pd complex and mostly reversed at 12 h (except for aspartate, histidine, lysine, and valine, whose levels remained altered). A strong DMA increase was a specific feature of Pd_2_Spm, also recovered at 12 h, the origin of which requires further investigation. The reported changes in ketone bodies seem to be a common feature of both drugs, reflecting enhanced energy requirements [15], although such does not lead to enhanced nucleotide levels as observed for cDDP [15]. In fact, the almost absent changes in nucleotides upon Pd_2_Spm exposure in liver tissue suggest a distinct mechanism of action for the two drugs, which particularly clear in liver cells. Interestingly, hepatic lipidic metabolism was hardly changed by Pd_2_Spm, with qualitative (weaker) similarities to cDDP, reversed at 12–48 h, suggesting enhanced biosynthesis of saturated FA.

Regarding breast tissue, here studied as a contribution for subsequent breast cancer studies, again it is observed that Pd_2_Spm impacts faster (1 h) than cDDP on polar metabolites, exhibiting similar metabolic features as reported for the latter [15] but is followed by faster recovery to control levels. However, the Pd complex specifically triggers DMA and DMSO_2_ increases as possible indicators of the indirect impact of changes in gut microflora and decreased levels of the short-chain fatty acid formate (probably with a similar origin but requiring further investigation). Markedly increased ADP levels may indicate enhanced energy requirements and, thus, ATP hydrolysis, in response to Pd_2_Spm, than with cDDP. As observed for the liver, the breast tissue lipidome responded weakly and with rapid recovery to control levels.

## 4. Materials and Methods

### 4.1. Chemicals

Cisplatin (cis-dichlorodiammine platinum (II), 99.9%), potassium tetrachloropalladate (II) (K_2_PdCl_4_, 98%) and spermine (N,N’-bis(3-aminopropyl)-1,4-diaminobutane, 99%) were purchased from Sigma-Aldrich (Sintra, Portugal). The Pd_2_Spm complex was synthesized according to published procedures [59] optimized by the authors [43]: 2 mmol of K_2_PdCl_4_ were dissolved in a small amount of water, and 1 mmol spermine aqueous solution was added dropwise under stirring. After 24 h, the resulting powder was filtered and washed with acetone (yield 68%). The newly synthesized compound was characterized (and tested as to purity) by elemental analysis and vibrational spectroscopy—Fourier transform infrared (FTIR) spectroscopy, Raman and inelastic neutron scattering (INS), and compared with previous data [43]. Elemental analysis, Found (for Pd_2_(C_10_N_4_H_26_)Cl_4_): C: 21.2%; H: 4.7%; N: 9.6%, Cl: 25.9%; Calculated—C: 21.5%; H: 4.6%; N: 9.9%, Cl: 25.6%. Euthasol^®^ solution (400 mg/mL pentobarbital sodium) was obtained from Le Vet (Oudewater, The Netherlands). All reagents were of analytical grade.

### 4.2. Ethical Considerations

The handling and care of animals were in full agreement with the Portuguese (Decreto-Lei n.◦113/2013) and European (Directive 2010/63/EU) legislation for the protection of animals used for scientific purposes and with the recommendations stated in the Guide for Care and Use of Laboratory Animals of the National Institutes of Health (NIH). The study protocol was approved by the Ethics Committee for Animal Experimentation of the Faculty of Pharmacy of the University of Porto, Porto, Portugal (Permit Number: 25-10-2015), and by the Ethics Committee and the Organ Responsible for the Welfare of Animals of ICBAS-UP, Porto, Portugal (Permit number 134/2015). The Animal Research: Reporting of In Vivo Experiments (ARRIVE) guidelines were followed [60].

### 4.3. Animals

Six-weeks old, Specific-Pathogen-Free (SPF), female BALB/cByJ mice (45 animals in total) were purchased from Charles River Laboratories (L’Arbresle, France) and acclimatized for 1 week at the ICBAS-UP Rodent Animal House Facility (Porto, Portugal). The animals were randomly distributed into groups of five per cage (individually ventilated) with enrichment material (corncob bedding, paper roll tube, and one large sheet of tissue paper for nesting). The animals were housed in a SPF environment with *ad libitum* access to water and standard pellet food under controlled 12 h light/dark cycles (lights on at 7.00 AM), temperature (22 ± 2 °C), and humidity (50 ± 10%).

### 4.4. In vivo Experimental Procedures

After 1 week of acclimatization, animals were randomly allocated into three groups (15 animals per group), to be injected with: Pd_2_Spm (3.0 mg/kg body weight, in 1% DMSO in phosphate-buffered saline solution, PBS), cDDP (3.5 mg/kg body weight, in PBS) or vehicle (PBS: H_2_PO_4_ 1.5 mM, Na_2_HPO_4_ 4.3 mM, KCl 2.7 mM, NaCl 150 mM, pH 7.4). At the beginning and end of the experiments, average animal weights were 20.1 ± 1.7 g and 20.3 ± 1.6g, respectively. Drugs and vehicle (PBS) were administered in single doses, via intraperitoneal injection (volume of injection = 200 µL). All injected solutions were sterile filtered. After drug/vehicle administration, five animals from each group were euthanized, at each time-point (1, 12, and 48 h), with pentobarbital intraperitoneal injection (120 mg/kg) followed by cardiac puncture (one control mouse developed inflammation symptoms for reasons unknown and was excluded from subsequent analysis). The left kidney, median liver lobe, and thoracic mammary glands were excised, snap frozen in liquid nitrogen, and stored at −80 °C until NMR analysis. No biochemical measurements were performed to detect kidney damage, as the volume of blood obtained was too small (<400 µL). However, no animals showed any weight loss, without any evidence of decreased food or drink intake or other behavioral changes, thus supporting the absence of significant kidney damage, as explained previously [15].

### 4.5. Sample Preparation for NMR Analysis

Tissues were weighted (ca. 50, 60, and 35 mg for kidney (superior half), liver (median liver lobe), and breast tissue (predominantly comprising mammary gland), respectively) and ground by mechanical maceration in liquid N_2_ [61,62,63]. During this procedure, one control kidney sample was lost, for technical reasons, therefore leaving 13 kidney control samples (instead of the expected 14). Tissue extracts were prepared by the biphasic methanol/chloroform/water (2.0:2.0:1.0) method [64]: samples were homogenized in cold 8.0 mL/g 80% methanol, 4.0 mL/g chloroform, and 2 mg/L water, vortexed for 60 s and kept on ice for 10 min [64]. Samples were centrifuged (8000 rpm, 5 min, 23 °C), and polar and nonpolar phases were removed into separate vials, vacuum-dried, and N_2_-jet dried respectively, before −80 °C storage. Before NMR analysis, aqueous extracts were suspended in 650 µL of 100 mM sodium phosphate buffer (pH 7.4, in D_2_O containing 0.25% 3-(trimethylsilyl)-propionic-2,2,3,3-d4 acid (TSP) for chemical shift referencing), and lipophilic extracts were suspended in 650 µL of CDCl_3_, containing 0.03% tetramethylsilane (TMS). Samples were homogenized, and 600 µL were transferred to 5mm NMR tubes.

### 4.6. NMR Spectroscopy

NMR spectra were acquired on a Bruker AVANCE III spectrometer operating at 500.13 MHz for ^1^H, at 298 K. The 1D proton NMR spectra of aqueous and lipophilic extracts were recorded using the “noesypr1d” and “zg” pulse sequences (Bruker library), respectively, with 2.34 s acquisition time, 2 s relaxation delay, 512 scans, 7002.801 Hz spectral width, and 32 k data points. Each free-induction decay was zero-filled to 64 k points and multiplied by a 0.3 Hz exponential function before Fourier transformation. Spectra were manually phased, baseline-corrected, and chemical shift referenced to TSP or TMS for aqueous and lipophilic extracts, respectively. 2D NMR homonuclear total correlation (TOCSY) and heteronuclear single-quantum correlation (HSQC) spectra were acquired for selected samples to aid spectral assignment, which was based on comparison with existing literature, and data available on the Bruker BIOREFCODE spectral database and the human metabolome database (HMDB) [65].

### 4.7. Data Processing and Statistics

1D NMR spectra were converted into matrices (AMIX-viewer 3.9.14, Bruker Biospin, Rheinstetten, Germany), after exclusion of the water (δ 4.5–5.2) and methanol (singlet at δ 3.36) regions for aqueous extracts, and of chloroform and corresponding satellites peaks (δ 6.89–7.55) for lipophilic extracts. Spectra were aligned by recursive, segment-wise peak alignment (RSPA) (Matlab 8.3.0, The MathWorks Inc., Natick, MA, USA) and normalized to the total spectral area. Multivariate analysis was carried out using principal component analysis (PCA) and partial least-squares discriminant analysis (PLS-DA) upon unit variance (UV) scaling (SIMCA-P 11.5; Umetrics, Umeå, Sweden). PLS-DA loadings were back-transformed, multiplying each variable by its standard deviation, and colored according to variable importance to the projection (VIP) (Matlab 8.3.0, The MathWorks Inc., Natick, MA, USA). PLS-DA models were taken as statistically robust for predictive power (Q^2^) values ≥ 0.05. The relevant resonances identified in PLS-DA loadings plots were integrated (Amix 3.9.5, Bruker BioSpin, Rheinstetten, Germany), normalized, and variations assessed by univariate analysis (Shapiro–Wilk test to assess data normality, Student’s *t*-test or Wilcoxon test for normally-distributed or non-normally distributed data, respectively) (R-statistical software). Significantly changed metabolites (*p* < 0.05) were identified, and the corresponding effect-size values were calculated [66]. False discovery rate (FDR) correction based on the Benjamini and Hochberg method [67] was used to correct *p*-values for multiple comparisons.

## 5. Conclusions

To the best of our knowledge, this paper describes the first NMR metabolomics study of different mice organs of healthy animals to test the metabolic impact and recovery dynamics of the potential anticancer drug Pd_2_Spm, compared to cisplatin. As well as recognizing specific metabolic signatures of the impact of the Pd complex on kidney, liver and breast tissue polar and lipophilic metabolomes (in spite of the limitation related to the lack of biochemical measurements of biotoxicity), two general ideas emerge: (1) pathways involving polar metabolites tend to respond more strongly and rapidly (within 1 h) to Pd_2_Spm than to cisplatin, than lipid metabolism which exhibits delayed (kidney) or weak responses (liver and breast tissue); (2) Pd_2_Spm triggers shorter-term responses and recovery times than cisplatin (with the exception of kidney lipid metabolism, which remains altered at 48 h), which may be suggestive of potential lower adverse effects of Pd_2_Spm effects and prompting it as a promising drug for clinical trials.

## Figures and Tables

**Table 1:** Predictive power (Q^2^) values corresponding to PLS-DA pairwise models obtained for spectra of aqueous and lipophilic extracts of kidney, liver, and breast tissue, corresponding to controls and each group of mice exposed to cDDP and Pd_2_Spm. Values in bold and with shading indicate a robust separation between controls and drug-exposed groups (Q^2^ ≥ 0.5), while enabling qualitative assessment of time-course dependences.

Tissue	Aqueous Extracts						Lipophilic Extracts					
	cDDP			Pd_2_Spm			cDDP			Pd_2_Spm		
	1 h	12 h	48 h	1 h	12 h	48 h	1 h	12 h	48 h	1 h	12 h	48 h
Kidney	**0.81**	**0.73**	**0.54**	**0.85**	0.46	**0.65**	**0.55**	0.37	**0.96**	0.45	**0.66**	**0.76**
Liver	0.42	**0.70**	**0.52**	**0.68**	**0.74**	0.33	**0.79**	<0	**0.65**	0.14	0.44	0.30
Breast tissue	<0	**0.54**	**0.54**	0.43	0.31	**0.59**	**0.59**	**0.54**	0.31	0.30	0.47	≈0

## Data Availability

Data available on request due to privacy restrictions.

## Supplementary Materials

The following are available online at https://www.mdpi.com/2218-1989/11/2/114/s1, Figure S1. Average 500 MHz ^1^H NMR spectra of lipophilic extracts of kidney, liver, and breast tissue.; Figure S2: Average 500 MHz ^1^H NMR spectra of lipophilic kidney extracts of mice exposed to Pd_2_Spm and cDDP; Figure S3: Average 500 MHz ^1^H NMR spectra of aqueous liver extracts of mice exposed to Pd_2_Spm and cDDP. Table S1. List of metabolite variations in aqueous kidney extracts of mice exposed to cDDP and Pd_2_Spm at 1, 12, and 48 h post-injection, compared to controls; Table S2. List of metabolite variations in aqueous liver extracts of mice exposed to cDDP and Pd_2_Spm at 1, 12, and 48h post-injection, compared to controls; Table S3. List of metabolite variations found in aqueous breast tissue extracts of mice exposed to cDDP and Pd_2_Spm at 1, 12, and 48 h post-injection, compared to controls; Table S4. List of metabolite variations found in lipophilic extracts of kidney, liver, and breast tissue of mice exposed to cDDP and Pd_2_Spm at 1, 12, and 48 h post-injection, compared to controls.
