# Supplementary material for: Novel Insights into Mice Multi-Organ Metabolism upon Exposure to a Potential Anticancer Pd(II)-Agent
Source: Metabolites. 2021 Feb 17;11(2):114. doi: 10.3390/metabo11020114 (PMC7922283; doi:10.3390/metabo11020114)
Supplement: Supplementary file 1 [file metabolites-11-00114-s001.pdf]

Supporting Information

Novel insights into mice multi-organ metabolism upon exposure to a potential anticancer Pd(II)-agent

Tatiana J. Carneiro[1], Rita Araújo[1], Martin Vojtek[2], Salomé Gonçalves-Monteiro[2], Carmen Diniz[2], Ana L.M. Batista de Carvalho[3], M. Paula M. Marques[3,4], Ana M. Gil[1,*]

[1] Department of Chemistry and CICECO – Aveiro Institute of Materials, University of Aveiro, 3810-193, Aveiro, Portugal;

[2] LAQV/REQUIMTE, Laboratory of Pharmacology, Department of Drug Sciences, Faculty of Pharmacy, University of Porto, 4050-313 Porto, Portugal;

[3] "Química-Física Molecular", University of Coimbra, 3004-535 Coimbra, Portugal;

[4] Department of Life Sciences, Faculty of Science and Technology, University of Coimbra, 3000-456 Coimbra, Portugal

* Correspondence: agil@ua.pt; Tel.: +351-234370707

Figure S1

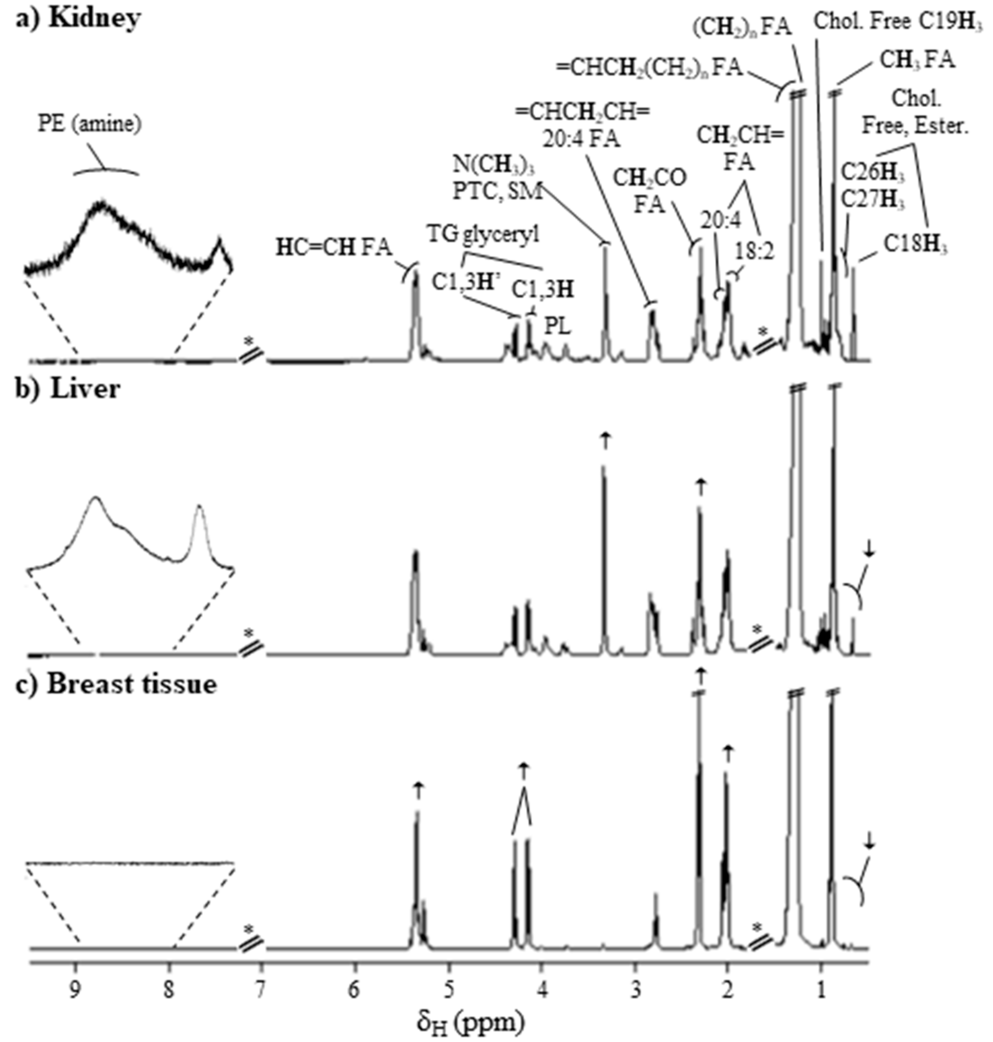

Figure S1. **Average 500 MHz ^{1}H NMR spectra of lipophilic extracts of kidney, liver and breast tissue**. Spectra obtained for (a) kidney, (b) liver and (c) breast tissue of BALB/c mice after 1h of post-injection with phosphate buffer saline (controls). * residual $CDCl_3$ (δ 7.15-7.37, and corresponding satellites at δ 7.04-7.06 and δ 7.45-δ7.47) and water (δ 1.45-1.70) regions, removed from matrix used for multivariate analysis. Arrows identify noticeable differences in some metabolites between tissues. Abbreviations: Chol., cholesterol; Ester., esterified; FA, fatty acid; PL, phospholipids glyceryl moieties; PTC, phosphatidylcholine; PE, phosphatidylethanolamine; SM, sphingomyelin; TG, triacylglyceride.

Figure S2

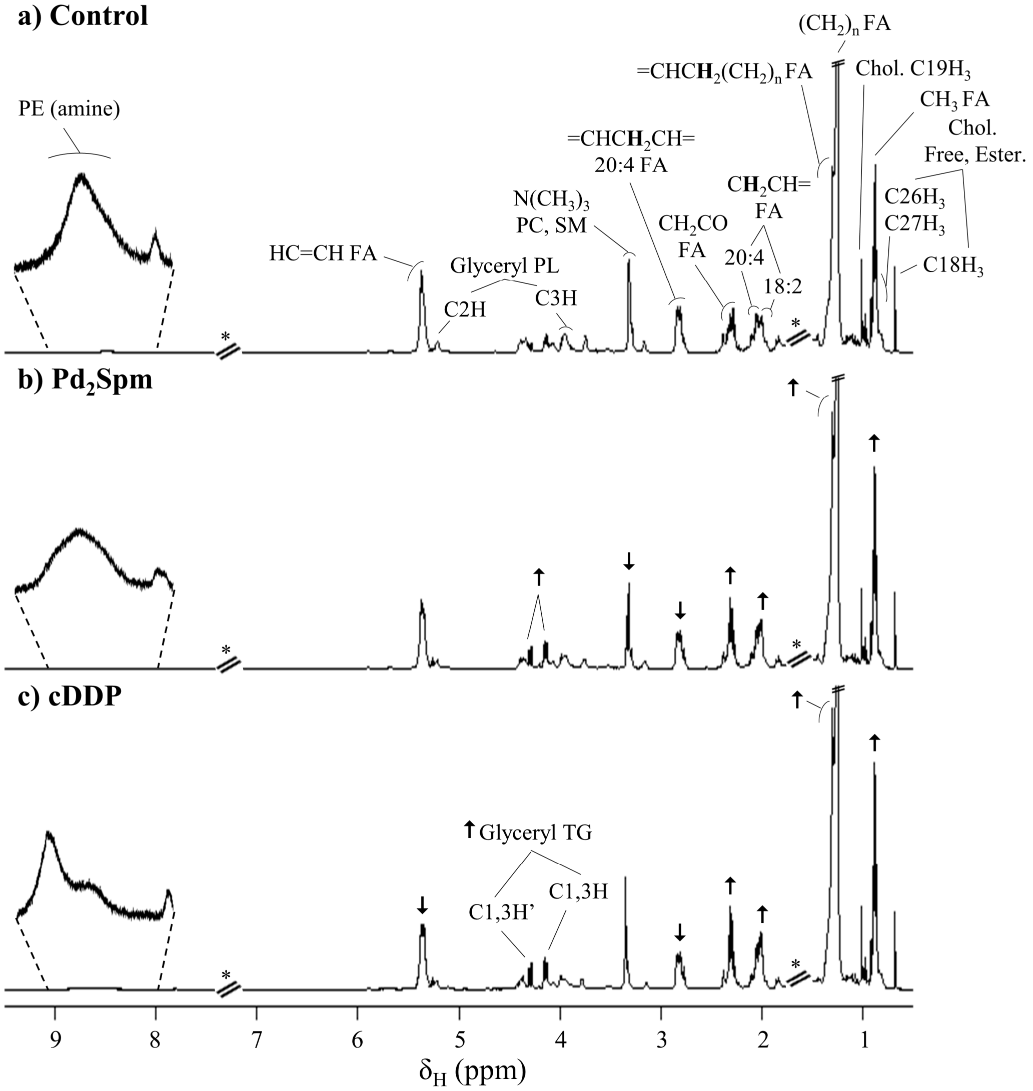

Figure S2. **Average 500 MHz ^{1}H NMR spectra of lipophilic kidney extracts of mice exposed to cDDP and Pd_2Spm.** Extracts correspond to BALB/c mice after 48 h of post-injection with (a) phosphate buffer saline (controls), (b) Pd_2Spm and (c) cDDP. * residual $CDCl_3$ (δ 7.15-7.37, and corresponding satellites at δ 7.04-7.06 and δ 7.45-δ7.47) and water (δ 1.50-1.90) regions, removed from matrix used for multivariate analysis. Abbreviations as defined in Figure S1.

Figure S3

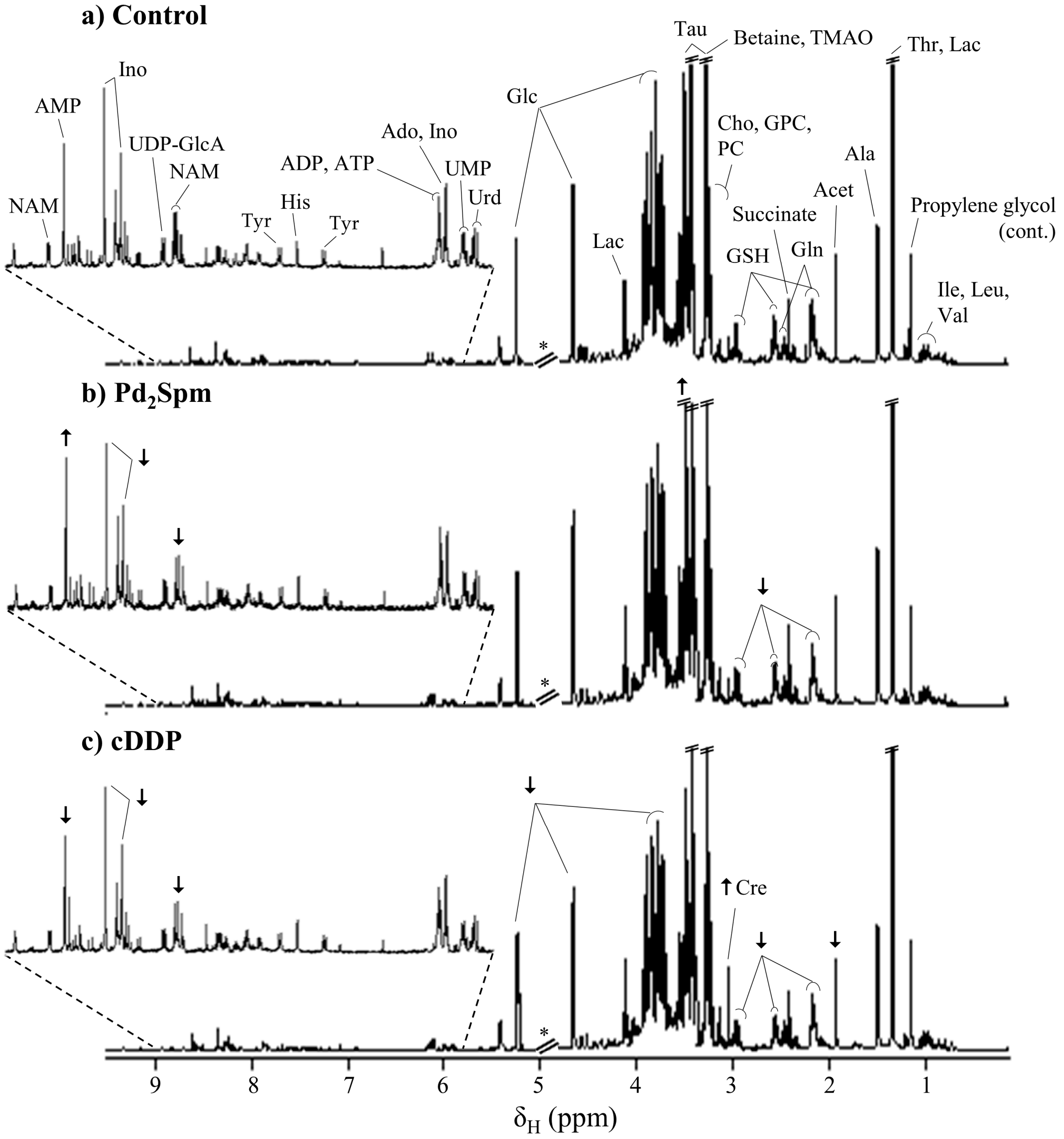

Figure S3. **Average 500 MHz ^{1}H NMR spectra of aqueous liver extracts of mice exposed to Pd_2Spm and cDDP**. Extracts were obtained for BALB/c mice after 12 h of post-injection with (a) phosphate buffer saline (controls), (b) Pd_2Spm and (c) cDDP. *Cut-off spectral region due to water suppression (δ4.69-5.10). Abbreviations: 3-letter code used for amino acids; Acet, acetate; Ado, adenosine; ADP, adenosine diphosphate; AMP, adenosine monophosphate; ATP, adenosine triphosphate; Cho, choline; Cre, creatine; Glc, glucose; GPC, glycerophosphocholine; GSH, reduced glutathione; Ino, inosine; Lac, lactate; NAM, niacinamide; PC, phosphocholine; Tau, taurine; TMAO, trimethylamine-*N*-oxide; UDP-GlcA: uridine diphosphate-glucuronate; UMP, uridine monophosphate; Urd, uridine.

Supplementary tables S1, S2, S3 and S4

Table S1. List of metabolite variations in aqueous kidney extracts of mice exposed to cDDP and Pd_2Spm at 1, 12 and 48 h post-injection, compared to controls. Only variations with 95% significance level (p-value < 0.05) are shown; ES, effect size; ES error values in brackets correspond to > 75% of ES value; [a] variations remaining significant after False Discovery Rate correction; [b] metabolic variations specific of exposure to Pd_2Spm (these comprise both new variations and variations in the same metabolite but in opposite directions). 3-letter code used for amino acids; 3-HIBA, 3-hydroxyisobutyrate; ADP, adenosine diphosphate; AMP, adenosine monophosphate; Cho, choline; Cre, creatine; DMA, dimethylamine; $DMSO_2$, dimethyl sulfone (tentative assignment); Glc, glucose; PC, phosphocholine; Tau, taurine; TMA, trimethylamine; NAD^+, nicotinamide adenine dinucleotide; UDP-GlcA, uridine-diphosphate-glucuronate; UMP, uridine monophosphate; Ui, unassigned i. † Tentative assignment. s: singlet, d: doublet, m: multiplet, t: triplet, dd: doublet of doublets.

Metabolite assignment	δ_H ppm multiplicity	**cDDP *vs.* Controls** (according to reference [15])						**Pd_2Spm *vs.* Controls**					
		1 h		12 h		48 h		1 h		12 h		48 h	
		ES ± Error	p-value	ES ± Error	p-value	ES ± Error	p-value	ES ± Error	p-value	ES ± Error	p-value	ES ± Error	p-value
3-HIBA	1.09 (d)	4.7 ± 2.5	1.9E-4 [a]	-2.2 ± 1.6	9.5E-3	- - -	-	2.7 ± 1.8	4.7E-3 [a]	- - -	-	- - -	-
ADP	8.28 (s)	1.8 ± (1.6)	2.9E-2	1.8 ± (1.5)	1.6E-2	- - -	-	2.3 ± 1.7	8.8E-3 [a]	- - -	-	- - -	-
Ala	1.48 (d)	-3.0 ± 1.9	9.5E-3 [a]	- - -	-	-2.2 ± 1.7	1.1E-2	-1.9 ± (1.6)	3.1E-2 [a]	- - -	-	- - -	-
Allantoin	5.39 (s)	- - -	-	-2.3 ± 1.6	9.3E-3	- - -	-	- - -	-	- - -	-	- - -	-
AMP	8.61 (s)	1.8 ± (1.5)	3.3E-2	1.9 ± (1.5)	2.8E-2	- - -	-	1.8 ± (1.6)	3.3E-2 [a]	- - -	-	- - -	-
Asn	2.96 (m)	- - -	-	-1.9 ± (1.5)	2.3E-2	- - -	-	- - -	-	- - -	-	- - -	-
Betaine	3.90 (s)	- - -	-	1.8 ± (1.5)	3.8E-2	- - -	-	- - -	-	- - -	-	- - -	-
Cho	3.21 (s)	-2.1 ± 1.6	1.6E-2	-3.2 ± 1.9	2.7E-3	- - -	-	-3.6 ± 2.1	8.2E-4 [a]	-2.7 ± 1.7	5.8E-3	- - -	-
Cre	3.93 (s)	- - -	-	-2.5 ± 1.6	1.1E-2	- - -	-	- - -	-	-2.3 ± 1.6	1.0E-2	-3.2 ± 2.0	1.1E-2
DMA	2.73 (s)	- - -	-	- - -	-	- - -	-	3.2 ± 2.0	5.5E-3 [a,b]	- - -	-	- - -	-
$DMSO_2$ †	3.15 (s)	- - -	-	-1.9 ± (1.5)	3.2E-2	- - -	-	9.8 ± 4.7	2.1E-5 [a, b]	- - -	-	- - -	-
Fumarate	6.52 (s)	- - -	-	- - -	-	-3.5 ± 2.1	2.7E-3 [a]	2.9 ± 1.9	2.1E-2 [a, b]	2.0 ± 1.5	1.4E-2 [b]	2.2 ± 1.7	1.5E-2
Glc	5.23 (d)	- - -	-	1.9 ± (1.5)	2.7E-2	- - -	-	- - -	-	- - -	-	- - -	-
Hypoxanthine	8.21 (s)	- - -	-	- - -	-	-2.6 ± 1.8	3.3E-2	- - -	-	- - -	-	- - -	-
Ile	1.01 (d)	-3.1 ± 1.9	4.1E-3 [a]	-1.9 ± (1.5)	3.3E-2	-2.2 ± 1.7	1.5E-2	-2.2 ± 1.7	1.2E-2 [a]	- - -	-	- - -	-
Leu	0.96 (t)	-3.5 ± 2.1	1.2E-3 [a]	-1.8 ± (1.5)	3.0E-2	-1.8 ± (1.6)	2.5E-2	-3.5 ± 2.1	1.5E-3 [a]	- - -	-	- - -	-
m-Inositol	4.06 (t)	- - -	-	- - -	-	- - -	-	-2.2 ± 1.7	1.3E-2 [a]	-1.7 ± (1.4)	3.1E-2 [b]	- - -	-

Niacinamide	7.60 (dd)	- - -	-	- - -	-	-1.7 ± (1.5)	3.6E-2	- - -	-	- - -	-	- - -	-
PC	3.22 (s)	- - -	-	1.9 ± (1.5)	1.7E-2	- - -	-	- - -	-	- - -	-	- - -	-
Phe	7.33 (d)	-3.2 ± 2.0	1.6E-2	- - -	-	- - -	-	-3.3 ± 2.0	1.6E-2 [a]	-1.6 ± (1.4)	3.6E-2	- - -	-
Succinate	2.41 (s)	- - -	-	- - -	-	- - -	-	2.0 ± (1.6)	2.0E-2 [a, b]	-1.5 ± (1.4)	4.3E-2 [b]	-1.6 ± (1.5)	3.2E-2 [b]
Tau	3.42 (t)	3.0 ± 1.9	3.2E-3 [a]	- - -	-	- - -	-	- - -	-	- - -	-	- - -	-
TMA	2.89 (s)	- - -	-	- - -	-	-4.3 ± 2.4	1.1E-3 [a]	- - -	-	- - -	-	- - -	-
Tyr	6.90 (d)	-2.1 ± 1.6	1.6E-2	- - -	-	-3.1 ± 1.9	2.5E-3 [a]	-2.5 ± 1.8	7.0E-3 [a]	- - -	-	- - -	-
NAD^+	8.43 (s)	- - -	-	1.7 ± (1.4)	1.6E-2	- - -	-	2.4 ± 1.7	1.6E-2 [a]	1.7 ± (1.5)	3.2E-2	- - -	-
UDP-GlcA	7.95 (d)	- - -	-	1.7 ± (1.4)	3.0E-2	- - -	-	2.9 ± 1.9	2.7E-3 [a]	- - -	-	-2.1 ± 1.6	2.8E-2 [b]
UMP	5.99 (m)	1.8 ± (1.6)	2.8E-2	- - -	-	- - -	-	2.2 ± 1.7	1.2E-2 [a]	- - -	-	- - -	-
Val	1.05 (d)	-2.5 ± 1.7	8.9E-3 [a]	-2.1 ± 1.5	2.0E-2	-2.0 ± (1.6)	2.1E-2	-2.2 ± 1.7	1.0E-2 [a]	- - -	-	- - -	-
U1	0.89 (t)	- - -	-	- - -	-	-2.5 ± 1.8	1.6E-2	- - -	-	1.9 ± (1.5)	1.9E-2 [b]	- - -	-
U2	0.93 (s)	- - -	-	-2.1 ± 1.6	1.2E-2	- - -	-	- - -	-	- - -	-	- - -	-
U3	1.62 (d)	- - -	-	-2.8 ± 1.7	5.0E-3	- - -	-	- - -	-	- - -	-	- - -	-
U4	2.92 (s)	- - -	-	-1.7 ± (1.4)	3.5E-2	- - -	-	3.5 ± 2.1	1.1E-3 [a, b]	- - -	-	2.1 ± 1.6	1.6E-2 [b]
U5	3.35 (s)	2.0 ± (1.6)	1.8E-2	1.8 ± (1.5)	2.2E-2	- - -	-	- - -	-	- - -	-	- - -	-

Table S2. **List of metabolite variations in aqueous liver extracts of mice exposed to cDDP and Pd_2Spm at 1, 12 and 48 h post-injection, compared to controls.** Only variations with 95% significance level (p-value < 0.05) are shown; ES, effect size; ES error values in brackets correspond to > 75% of ES value; [a] variations remaining significant after False Discovery Rate correction.; [b] metabolite variations specific of exposure to Pd_2Spm (these comprise both new variations and variations in the same metabolite but in opposite directions). † Tentative assignment. 3-HBA, 3-hydroxybutyrate; Ado, adenosine; ATP, adenosine triphosphate; GPC, glycerophosphocholine; GSH, glutathione (reduced); IMP, inosine monophosphate; Ino, inosine; other abbreviations as defined in Table S1.

**Metabolite assignment**	**δ_H ppm multiplicity**	**cDDP *vs.* Controls** (according to reference [15])						**Pd_2Spm *vs.* Controls**					
		1 h		12 h		48 h		1 h		12 h		48 h	
		ES ± Error	p-value	ES ± Error	p-value	ES ± Error	p-value	ES ± Error	p-value	ES ± Error	p-value	ES ± Error	p-value
2-aminobutyrate †	0.80 (t)	- - -	-	-1.6 ± (1.4)	4.4E-2	- - -	-	-1.5 ± (1.4)	3.7E-2 [a]	- - -	-	- - -	-
3-HBA	1.20 (d)	- - -	-	-1.8 ± (1.5)	3.1E-2	- - -	-	-1.5 ± (1.4)	4.8E-2	- - -	-	- - -	-
Acetate	1.92 (s)	- - -	-	- - -	-	-2.5 ± 1.7	2.0E-2	- - -	-	- - -	-	-1.7 ± (1.5)	4.1E-2
Acetone	2.24 (s)	- - -	-	-5.1 ± 2.6	5.2E-5 [a]	- - -	-	- - -	-	-1.9 ± (1.6)	3.7E-5 [a]	- - -	-
Ado/ Ino	4.44 (dd)	- - -	-	- - -	-	- - -	-	-1.8 ± (1.5)	2.0E-2 [b]	- - -	-	- - -	-
ADP	8.54 (s)	- - -	-	- - -	-	2.6 ± 1.8	1.6E-2	- - -	-	- - -	-	- - -	-
AMP	4.51 (dd)	- - -	-	- - -	-	2.8 ± 1.8	3.8E-3	- - -	-	- - -	-	- - -	-
Asp	2.67 (dd)	- - -	-	- - -	-	- - -	-	- - -	-	- - -	-	-2.0 ± (1.6)	3.6E-2 [b]
ATP	8.52 (s)	- - -	-	- - -	-	2.3 ± 1.7	1.6E-2	- - -	-	- - -	-	- - -	-
DMA	2.73 (s)	- - -	-	-2.4 ± 1.6	6.0E-3 [a]	- - -	-	3.2 ± 1.9	6.9E-3 [a,b]	- - -	-	- - -	-
Formate	8.46 (s)	- - -	-	-1.9 ± (1.5)	7.9E-3 [a]	- - -	-	- - -	-	- - -	-	- - -	-
GPC	3.23 (s)	- - -	-	- - -	-	- - -	-	- - -	-	1.6 ± (1.5)	2.1E-2 [b]	- - -	-
GSH	2.55 (m)	- - -	-	-4.0 ± 2.1	2.7E-4 [a]	- - -	-	- - -	-	- - -	-	- - -	-
His	7.08 (s)	- - -	-	2.2 ± 1.6	9.1E-3	- - -	-	- - -	-	- - -	-	1.6 ± (1.5)	4.3E-2
Ile	1.01 (d)	- - -	-	- - -	-	- - -	-	-2.3 ± 1.6	5.8E-3 [a,b]	- - -	-	- - -	-
IMP	8.58 (s)	- - -	-	- - -	-	1.7 ± (1.5)	3.2E-2	- - -	-	- - -	-	- - -	-
Ino	8.35 (s)	- - -	-	- - -	-	- - -	-	-1.7 ± (1.5)	2.5E-2 [a,b]	- - -	-	- - -	-
Lactate	4.10 (q)	-2.7 ± 1.7	6.9E-3	1.7 ± (1.5)	3.0E-2	- - -	-	- - -	-	- - -	-	- - -	-
Leu	0.96 (t)	- - -	-	- - -	-	- - -	-	-2.0 ± 1.5	8.7E-3 [a,b]	- - -	-	- - -	-
Lys	1.71 (m)	- - -	-	- - -	-	- - -	-	-1.8 ± (1.5)	1.3E-2 [a,b]	- - -	-	2.6 ± 1.8	5.5E-3 [b]
NAD^+	8.43 (s)	- - -	-	- - -	-	2.6 ± 1.8	9.9E-3	- - -	-	- - -	-	- - -	-

Phe	7.33 (d)	-	-	-	-	-	-	-	-	-	-	-	-	-1.6	±	(1.4)	2.2E-2 [a,b]	-	-	-	-	-	-	-	-
Thr, Lactate	1.33 (d)	-2.3	±	1.6	3.2E-2	1.9	±	(1.5)	1.9E-2	-	-	-	-	-	-	-	-	-	-	-	-	-	-	-	-
Tyr	6.90 (d)	-	-	-	-	-	-	-	-	1.9	±	(1.6)	2.3E-2	-1.7	±	(1.4)	1.9E-2 [a,b]	-	-	-	-	-	-	-	-
UDP-GlcA	7.95 (d)	-	-	-	-	-1.7	±	(1.4)	3.0E-2	-	-	-	-	-	-	-	-	-	-	-	-	-	-	-	-
Uridine	7.86 (d)	-	-	-	-	-	-	-	-	-	-	-	-	-2.6	±	1.7	2.4E-3 [a,b]	-	-	-	-	-	-	-	-
Val	1.05 (d)	-	-	-	-	-	-	-	-	-	-	-	-	-1.9	±	(1.5)	1.1E-2 [a,b]	-	-	-	-	1.9	±	(1.6)	2.3E-2 [b]
U6	0.73 (s)	-2.2	±	1.6	1.6E-2	-	-	-	-	2.6	±	1.8	1.6E-2	-	-	-	-	-	-	-	-	-	-	-	-
U7	0.85 (t)	-	-	-	-	-1.6	±	(1.4)	1.6E-2	-	-	-	-	-	-	-	-	-	-	-	-	-	-	-	-
U8	3.10 (d)	-	-	-	-	2.5	±	1.7	9.8E-3 [a]	-	-	-	-	2.5	±	1.7	8.4E-3 [a]	-	-	-	-	-	-	-	-
U9	4.31 (d)	-1.6	±	(1.4)	4.7E-2	-1.8	±	(1.5)	2.1E-2	1.7	±	(1.5)	3.3E-2	-	-	-	-	-	-	-	-	-	-	-	-
U10	6.18 (s)	-1.7	±	(1.5)	3.6E-2	-2.6	±	1.7	7.8E-3 [a]	2.2	±	1.7	2.3E-2	-	-	-	-	-	-	-	-	-	-	-	-
U11	8.28 (br)	-	-	-	-	-1.8	±	(1.5)	3.5E-2	1.8	±	(1.5)	4.2E-2	-	-	-	-	-	-	-	-	-	-	-	-

Table S3. List of metabolite variations found in aqueous breast tissue extracts of mice exposed to cDDP and Pd_2Spm at 1, 12 and 48 h post-injection, compared to controls. Only variations with 95% significance level (p-value < 0.05) are shown; ES, effect size; ES error values in brackets correspond to > 75% of ES value; [a] variations remaining significant after False Discovery Rate correction, [b] metabolite variations specific of exposure to Pd_2Spm (these comprise both new variations and variations in the same metabolite but in opposite directions). † Tentative assignment. Abbreviations as defined in Tables S1 and S2.

Metabolite assignment	δ_H ppm multiplicity	**cDDP *vs.*Controls** (according to reference [15])						**Pd_2Spm *vs.*Controls**					
		1 h		12 h		48 h		1 h		12 h		48 h	
		ES ± Error	p-value	ES ± Error	p-value	ES ± Error	p-value	ES ± Error	p-value	ES ± Error	p-value	ES ± Error	p-value
3-HBA	1.20 (d)	1.8 ± (1.5)	4.3E-2	- - -	-	- - -	-	- - -	-	- - -	-	- - -	-
Ado/Ino	6.10 (d)	- - -	-	-2.4 ± 1.6	6.1E-3 [a]	- - -	-	- - -	-	-1.9 ± (1.5)	1.5E-2	- - -	-
ADP	8.54 (s)	- - -	-	1.7 ± (1.4)	3.1E-2 [a]	- - -	-	1.9 ± (1.5)	1.6E-2	4.5 ± 2.3	1.8E-4 [a]	- - -	-
Creatinine	3.04 (s)	- - -	-	- - -	-	- - -	-	- - -	-	- - -	-	1.6 ± (1.5)	3.2E-2 [b]
DMA	2.73 (s)	- - -	-	- - -	-	- - -	-	3.6 ± 2.0	4.9E-3 [a,b]	- - -	-	- - -	-
$DMSO_2$ †	3.15 (s)	- - -	-	- - -	-	- - -	-	8.8 ± 4.0	1.8E-6 [a,b]	- - -	-	- - -	-
Formate	8.46 (s)	- - -	-	- - -	-	- - -	-	- - -	-	-1.6 ± (1.4)	4.7E-2 [b]	- - -	-
Gln	2.45 (m)	- - -	-	- - -	-	-2.2 ± 1.6	1.2E-2	- - -	-	- - -	-	- - -	-
Gly	3.56 (s)	- - -	-	- - -	-	- - -	-	-1.6 ± (1.4)	4.6E-2 [b]	- - -	-	- - -	-
GSH	2.55 (m)	- - -	-	- - -	-	- - -	-	1.8 ± (1.5)	2.0E-2 [b]	- - -	-	- - -	-
Guanine †	7.68 (s)	- - -	-	- - -	-	-1.9 ± (1.6)	3.1E-2	- - -	-	-1.9 ± (1.5)	2.5E-2	-1.8 ± (1.6)	3.2E-2
Ino	8.35 (s)	- - -	-	-2.8 ± 1.7	2.4E-3 [a]	- - -	-	- - -	-	-2.3 ± 1.6	6.8E-3 [a]	- - -	-
Tyr	6.90 (d)	- - -	-	- - -	-	- - -	-	- - -	-	-1.6 ± (1.4)	3.7E-2 [b]	- - -	-
U12	1.25 (s)	- - -	-	2.0 ± 1.5	2.6E-2 [a]	- - -	-	2.1 ± 1.6	1.7E-2	2.0 ± 1.5	2.4E-2	- - -	-

Table S4. List of metabolite variations found in lipophilic extracts of kidney, liver and breast tissue of mice exposed to cDDP and Pd_2Spm at 1, 12 and 48 h post-injection, compared to controls. Only variations with 95% significance level (p-value < 0.05) are shown; ES, effect size; ES error values in brackets correspond to > 75% of ES values; [a] variations remaining significant after False Discovery Rate correction, [b] metabolite variations specific of exposure to Pd_2Spm (these comprise both new variations and variations in the same metabolite but in opposite directions).

Metabolite family/ assignment		δ_H ppm multiplicity	cDDP *vs.* Controls						Pd_2Spm *vs.* Controls					
			1 h		12 h		48 h		1 h		12 h		48 h	
			ES ± Error	p-value	ES ± Error	p-value	ES ± Error	p-value	ES ± Error	p-value	ES ± Error	p-value	ES ± Error	p-value
**KIDNEY**														
Cholesterol	Free/ Esterified, C18H_3	0.68 (s)	1.9 ± (1.5)	2.0E-2 [a]	- - -	-	- - -	-	- - -	-	- - -	-	- - -	-
	Free, C4**H'**	2.27 (t)	1.7 ± (1.4)	3.2E-2 [a]	- - -	-	- - -	-	- - -	-	- - -	-	- - -	-
	Esterified, C4**H'**	2.31 (t)	1.7 ± (1.5)	2.5E-2 [a]	- - -	-	-2.9 ± 1.9	7.7E-3 [a]	- - -	-	- - -	-	- - -	-
	Free, C3**H**	3.53 (m)	- - -	-	- - -	-	- - -	-	- - -	-	- - -	-	-1.6 ± (1.5)	4.2E-2 [b]
Fatty acids	CH_3	0.89 (br)	- - -	-	- - -	-	1.7 ± (1.5)	4.4E-2	- - -	-	- - -	-	2.1 ± (1.6)	1.7E-2
	$=CHCH_2(CH_2)_n$	1.30 (br)	-1.6 ± (1.4)	3.2E-2 [a]	- - -	-	- - -	-	- - -	-	- - -	-	1.7 ± (1.5)	4.3E-2 [b]
	CH_2CO	2.30 (m)	-1.6 ± (1.4)	3.7E-2 [a]	- - -	-	- - -	-	- - -	-	- - -	-	1.6 ± (1.5)	4.9E-2 [b]
	18:2 $=CHCH_2CH=$	2.77 (t)	-1.6 ± (1.4)	3.2E-2 [a]	- - -	-	- - -	-	- - -	-	- - -	-	- - -	-
	18:2 **HC=CH**	5.34 (m)	- - -	-	- - -	-	- - -	-	- - -	-	- - -	-	1.7 ± (1.5)	1.6E-2 [b]
	18:2/ 20:4 CH_3	0.98 (t)	1.5 ± (1.4	4.4E-2	- - -	-	- - -	-	- - -	-	- - -	-	-1.8 ± (1.6)	2.7E-2 [b]
	20:4 $=CHCH_2CH=$	2.81 (m)	- - -	-	- - -	-	- - -	-	- - -	-	- - -	-	-1.6 ± (1.5)	4.9E-2 [b]
	20:4 **HC=CH**	5.37 (m)	1.6 ± (1.4)	3.2E-2 [a]	- - -	-	- - -	-	- - -	-	- - -	-	- - -	-
Phospholipids	$CH_2N(CH_3)_3$	3.75 (br)	1.8 ± (1.5)	2.4E-2 [a]	- - -	-	-2.6 ± 1.8	7.9E-3 [a]	- - -	-	- - -	-	-1.6 ± (1.5)	4.7E-2
	Glyceryl C3H_2	3.94 (br)	2.5 ± 1.7	4.6E-3 [a]	- - -	-	-3.9 ± 2.2	7.0E-4 [a]	- - -	-	- - -	-	- - -	-
	Glyceryl C1H_2	4.38 (br)	- - -	-	- - -	-	- - -	-	- - -	-	- - -	-	-1.6 ± (1.5)	5.0E-2 [b]
	Glyceryl C2**H**	5.20 (br)	2.0 ± 1.5	3.2E-2 [a]	- - -	-	-2.1 ± 1.6	2.0E-2	- - -	-	- - -	-	- - -	-
	PE CH_2NH_3	3.16 (br)	- - -	-	- - -	-	-1.8 ± (1.5)	1.6E-2	- - -	-	- - -	-	-1.8 ± (1.6)	2.8E-2
	PTC/SM, $N(CH_3)_3$	3.29/3.31 (s)	1.7 ± (1.4)	3.2E-2 [a]	- - -	-	-1.6 ± (1.5)	5.0E-2	- - -	-	- - -	-	-1.7 ± (1.5)	3.5E-2
Triglycerides	Glyceryl C1, **3H**	4.14 (dd)	- - -	-	- - -	-	- - -	-	- - -	-	- - -	-	1.8 ± (1.6)	2.9E-2 [b]
	Glyceryl C2**H**	5.26 (m)	-1.8 ± (1.5)	2.3E-2 [a]	- - -	-	1.7 ± (1.5)	4.3E-2	- - -	-	- - -	-	- - -	-
Lipid ratios	$(CH_2)_n/CH_3$	-	- - -	-	- - -	-	- - -	-	- - -	-	- - -	-	-1.6 ± (1.5)	4.9E-2 [b]
	$HC=CH/CH_3$	-	- - -	-	- - -	-	- - -	-	- - -	-	- - -	-	-1.7 ± (1.5)	3.2E-2 [b]

Unknowns	U1	0.82 (br)	- - -	-	- - -	-	- - -	-	-3.0 ± 1.8	4.4E-2 [b]	- - -	-	- - -	-
	U2	2.38 (br)	- - -	-	- - -	-	- - -	-	- - -	-	- - -	-	-1.8 ± (1.6)	3.1E-2 [b]
	U3	4.07 (br)	1.9 ± (1.5)	1.9E-2 [a]	- - -	-	- - -	-	- - -	-	- - -	-	-1.6 ± (1.5)	3.9E-2 [b]
	U4	4.20 (dd)	- - -	-	- - -	-	- - -	-	- - -	-	11.1 ± 5.0	1.7E-7 [a,b]	2.7 ± 1.8	6.5E-3 [b]
	U5	4.69 (d)	- - -	-	- - -	-	- - -	-	- - -	-	- - -	-	-2.7 ± 1.8	3.9E-3 [b]
	U6	5.90 (d)	- - -	-	- - -	-	-1.9 ± (1.6)	2.6E-2	- - -	-	- - -	-	-1.9 ± (1.6)	2.3E-2
	U7	7.88 (br)	1.8 ± (1.5)	2.7E-2 [a]	- - -	-	-3.1 ± 2.0	2.6E-3 [a]	- - -	-	- - -	-	-2.1 ± (1.6)	3.2E-2
**LIVER**														
Cholesterol	Free/ Esterified, $C26\mathbf{H}_3$	0.86 (d)	- - -	-	- - -	-	1.6 ± (1.5)	4.3E-2	- - -	-	- - -	-	- - -	-
	Free, $C19\mathbf{H}_3$	1.01 (s)	- - -	-	- - -	-	- - -	-	- - -	-	1.6 ± (1.4)	4.5E-2 [b]	- - -	-
Fatty acids	$\mathbf{CH}_3$	0.89 (br)	- - -	-	- - -	-	- - -	-	- - -	-	2.0 ± 1.5	3.0E-2 [b]	- - -	-
	$(\mathbf{CH}_2)_n$	1.25 (br)	- - -	-	- - -	-	- - -	-	- - -	-	2.1 ± 1.6	1.7E-2 [b]	- - -	-
	18:2/ 20:4 $\mathbf{CH}_3$	0.98(t)	- - -	-	- - -	-	2.4 ± 1.7	6.9E-3 [a]	- - -	-	- - -	-	- - -	-
	20:4 $\mathbf{CH}_2$CH=	2.07 (q)	-3.2 ± 2.0	1.9E-2 [a]	- - -	-	- - -	-	-2.2 ± 1.7	1.6E-2 [b]	- - -	-	- - -	-
	20:4 =CH$\mathbf{CH}_2$CH=	2.81 (m)	-2.2 ± 1.7	1.7E-2 [a]	- - -	-	- - -	-	- - -	-	- - -	-	- - -	-
	20:4 **HC=CH**	5.37 (m)	-2.4 ± 1.7	1.3E-2 [a]	- - -	-	- - -	-	-1.6 ± (1.5)	3.7E-2	- - -	-	- - -	-
	22:6 $\mathbf{CH}_2$CO	2.37 (br)	-2.2 ± 1.7	4.6E-2	- - -	-	1.9 ± (1.6)	2.9E-2	-1.9 ± (1.6)	4.1E-2	- - -	-	- - -	-
**BREAST TISSUE**														
Cholesterol	Free/ Esterified, C3**H**	1.02 (s)	- - -	-	-1.7 ± (1.4)	4.0E-2 [a]	- - -	-	-1.8 ± (1.5)	3.2E-2	- - -	-	- - -	-
Fatty Acids	$\mathbf{CH}_3$	0.89 (br)	1.7 ± (1.4)	3.0E-2	- - -	-	- - -	-	- - -	-	- - -	-	- - -	-
	$(\mathbf{CH}_2)_n$	1.25 (br)	- - -	-	-2.7 ± 1.7	4.7E-3 [a]	- - -	-	- - -	-	-1.7 ± (1.5)	3.0E-2 [a]	- - -	-
	=CH$\mathbf{H}_2(CH_2)_n$	1.30 (br)	- - -	-	2.2 ± 1.6	1.2E-2 [a]	- - -	-	- - -	-	- - -	-	- - -	-
	$\mathbf{CH}_2$CO	2.30 (m)	- - -	-	1.8 ± (1.5)	3.2E-2 [a]	- - -	-	- - -	-	- - -	-	- - -	-
	18:2, **HC=CH**	5.34 (m)	- - -	-	2.9 ± 1.8	2.9E-3 [a]	- - -	-	- - -	-	1.8 ± (1.5)	2.2E-2 [a]	- - -	-
	18:2, =CH$\mathbf{CH}_2$CH=	2.77 (t)	- - -	-	2.2 ± 1.6	8.1E-3 [a]	- - -	-	- - -	-	1.6 ± (1.4)	1.6E-2 [a]	- - -	-
	20:4, **HC=CH**	5.36 (m)	- - -	-	2.3 ± 1.6	6.9E-3 [a]	- - -	-	- - -	-	1.7 ± (1.4)	3.3E-2 [a]	- - -	-
	20:4, $\mathbf{CH}_2$CH=	2.07 (q)	- - -	-	2.2 ± 1.6	8.8E-3 [a]	- - -	-	- - -	-	1.5 ± (1.4)	4.7E-2	- - -	-
Triglycerides	Glyceryl C1, **3H**	4.14 (dd)	- - -	-	1.7 ± (1.4)	3.9E-2 [a]	- - -	-	- - -	-	- - -	-	- - -	-
	Glyceryl C1, **3H'**	4.29 (dd)	- - -	-	1.8 ± (1.5)	2.9E-2 [a]	- - -	-	- - -	-	- - -	-	- - -	-
	Glyceryl C2**H**	5.26 (m)	- - -	-	4.4 ± 2.3	1.3E-4 [a]	- - -	-	1.5 ± (1.4)	4.8E-2	2.6 ± 1.7	4.3E-3 [a]	- - -	-
Lipid ratios	$(\mathbf{CH}_2)_n/CH_3$	-	-1.7 ± (1.4)	4.5E-2	-1.7 ± 1.4	2.9E-2 [a]	- - -	-	- - -	-	-2.1 ± 1.5	1.3E-2 [a]	- - -	-
	$\mathbf{HC{=}CH}/CH_3$	-	-1.8 ± (1.5)	3.7E-2	- - -	-	- - -	-	- - -	-	-1.6 ± (1.4)	3.7E-2 [a]	- - -	-
Unknows	U4	4.20 (dd)	- - -	-	-5.0 ± 2.5	3.4E-4 [a]	-3.2 ± 2.0	2.3E-2	-1.9 ± (1.5)	2.0E-2 [a]	-4.3 ± 2.3	8.1E-4 [a]	- - -	-

U8	2.17 (s)	3.1 ± 1.8	1.5E-3 [a]	- - -	-	- - -	-	- - -	-	- - -	-	- - -	-
U9	3.60 (dd)	3.0 ± 1.8	1.8E-3 [a]	-5.3 ± 2.6	3.0E-4 [a]	-6.6 ± 3.3	2.4E-3 [a]	-3.2 ± 1.9	2.3E-3 [a]	-5.1 ± 2.5	9.3E-4 [a]	-6.0 ± 3.1	1.3E-3 [a]
U10	3.64 (s)	- - -	-	-4.0 ± 2.1	4.3E-4 [a]	-4.0 ± 2.3	1.6E-2	-2.3 ± 1.6	1.5E-2 [a]	-4.0 ± 2.1	2.4E-3 [a]	- - -	-
U11	4.38 (br)	- - -	-	-1.7 ± (1.4)	3.8E-2 [a]	- - -	-	- - -	-	- - -	-	- - -	-
